# Equity across the cancer care continuum for culturally and linguistically diverse migrants living in Australia: a scoping review

**DOI:** 10.1186/s12992-021-00737-w

**Published:** 2021-07-28

**Authors:** Brighid Scanlon, Mark Brough, David Wyld, Jo Durham

**Affiliations:** 1 Queensland University of Technology, 149 Victoria Park Road, QLD 4059 Kelvin Grove, Australia; 2 Royal Brisbane and Women’s Hospital, Butterfield Street, QLD 4029 Herston, Australia; 3 University of Queensland, 20 Weightman Street, QLD 4006 Herston, Australia

**Keywords:** Cancer, Disparities, Racialisation, Health Equity, Migrants, Culturally Diverse

## Abstract

International evidence suggests migrants experience inequitable access, outcomes and treatment quality across the cancer care continuum. There is currently limited research assessing equity across the cancer care continuum for culturally and linguistically diverse migrants living in Australia. A detailed protocol and search strategy were developed and used to identify all relevant literature, utilising the Joanna Briggs Institute Reviewer’s Manual. Systematic searching was conducted via multiple databases and identified studies were screened against pre-identified inclusion and exclusion criteria. 71 studies met the inclusion criteria for analysis. Most studies examined cancer detection via screening. Very few studies examined cancer prevention, diagnosis, treatment or palliative care. Most studies focused on patient-sided barriers to care and there was a paucity of information regarding institutional barriers to health. Cancer-related outcomes were seldom examined, and most studies were qualitative or behavioral analysis. Results highlighted significant communication issues spanning the cancer care continuum and a context of inadequate support for both patients and clinicians. There is a demonstrable need to examine equity in access and outcomes for culturally and linguistically diverse cancer populations. This requires the identification of cancer-related disparities and an examination of institutional barriers to care. Through addressing this dearth of information, future research and health policy can support the operationalisation of health equity.

## Background

Global migration continues to play an important role in human history and is often influenced by periods of instability. Instability arises from factors such as weak governance, unequal distribution of resources, violent conflict, social injustice, persistent negative impact from disasters, including pandemics such as COVID-19, economic hardship and poverty [1, 2]. Currently, over 272 million migrants have sought safety or opportunity in new countries and states, influencing the cultural and linguistic diversity of many regions [3]. As the COVID-19 pandemic demonstrates, disruptive events can expose existing inequities, or create new inequities, in areas such as health. Migrants from culturally and linguistically diverse (CALD) backgrounds may be particularly impacted, as the health systems they find themselves reliant on are often based on a culturally dominant model of care [4]. This approach is not responsive to the needs of diverse or minority populations and thus can produce and reproduce health inequities. It is therefore imperative that multilingual and multi-ethnic societies, such as Australia, strive for the promotion of health equity for all individuals [5]. Whilst the term ‘CALD’ is common within the Australian literature, the most consistent term used in the international literature is ‘ethnic minority’. Due to this, both terms were examined within this review. This research was conducted with consideration of the Australian historical backdrop of invasion, colonisation and dispossession of Aboriginal and Torres Strait Islander peoples. Thus, terminology such as ‘migrant’, ‘ethnic minority’ and ‘CALD’ have been utilised with caution and respect.

Health disparities exist both within and between countries [6] and in a globalised world, it is imperative to consider the health effects of differing disease burdens on migrant populations. Within countries of the Organization for Economic Co-operation and Development (OECD) and emerging economies, demographic and epidemiological transitions are underway, characterised by an aging population and a high burden of non-communicable diseases; including cancer [7–9]. A systematic analysis of 195 countries displayed that between the years 2006 and 2016, incident cases for all cancers increased significantly [7]. However, the burden of certain cancer types differs greatly between countries, for example cancers with infectious etiologies such as cervical, liver and gastric cancers are overrepresented in those from lower-middle income countries [7, 10]. In addition, effective cancer detection and treatment requires significant resource allocations and sophisticated diagnostic and therapeutic services and thus vary greatly between countries [11]. These factors influence health disparities between countries and must be considered when providing equitable and responsive cancer care in the post-migratory context [12].

Within high-income countries inequities across the cancer care continuum are a pertinent exemplar of the widening of health disparities for CALD populations [13]. The chronicity and complexity of the cancer journey makes it a valuable measure for health disparity research, with disparities in access and outcomes being mirrored in other chronic diseases affecting ethnic minority populations, such as cardiovascular disease and diabetes mellitus [14]. Research from the United States has displayed significant disparities across the cancer care continuum for ethnic minority populations, including in African American, Asian American, Latino/Hispanic and Pacific Islander populations [15]. In the United States, cancer is the leading cause of death for Latino people, with Latino women experiencing the highest cervical cancer rates, with incidence 64 % higher than ‘non-Hispanic white women’ [15]. American-Samoan men are eight-times more likely to develop liver cancer and American-Samoan women are twice as likely to develop, and die, from cervical cancer than ‘non-Hispanic white women’ [15]. Canadian research also demonstrates disparities for ethnic minority populations in access to screening, follow-up of abnormal findings, length of survival, quality of life, adherence to treatment regimens and quality of interactions with physicians [16, 17].

Health disparities have been defined as,…a particular type of health difference closely linked with economic, social, or environmental disadvantage. Health disparities adversely affect groups of people who have systematically experienced greater social or economic obstacles to health…[18].

Disparities in cancer care that disproportionately affect racialised groups are no longer considered the result of biological or behavioural mechanisms, but of socially and politically constructed identities that reflect power differentials and discrimination throughout society [19]. The powerful effects of these processes create macro societal and micro institutional barriers, which can manifest as institutional racism, fragmented health systems that are challenging to navigate, a lack of appropriate and accessible health information, high out-of-pocket expenditure and an environment of poor communication [13, 20, 21]. Despite this, health disparity research continues to perpetuate an individualistic, deficit perspective that fails to acknowledge the structural and institutional drivers of inequity.

Historically, the reduction of health disparities has been the predominant focus of research, however, recently attention has turned to promoting equity [4, 22]. Health equity has been defined as “…the principle underlying a commitment to reduce, and ultimately eliminate disparities in health and in its determinants” [23]. A useful way to examine equity throughout the multiple stages of cancer is to utilise the cancer care continuum framework. Developed in the 1970 s, this framework describes the various stages of cancer care, including prevention, detection, diagnosis, treatment and survivorship [24]. An important omission of the framework is its lack of attention to end-of-life or palliative care, which will be included in this review as an additional stage of the cancer care continuum. To date, whilst there has been some research into cancer-related health disparities for CALD populations in Australia, there is a distinct lack of attention to the diverse and changing needs of populations across the cancer care continuum. This is significant, as Australia is a multi-ethnic and multilingual society and therefore health services must adapt to the changing needs of populations.

The intent of this review is to synthesise what is currently known about equity across the cancer care continuum for CALD migrant populations in Australia and to identify areas in need of further research. This national focus acknowledges that whilst the needs of specific populations may differ, there is a need to develop health services that are adaptive and responsive to the changing needs of populations.

## Main Text

### Literature review process

A scoping review was deemed the most appropriate method, due to the scarcity of high-quality published information on the topic. This allowed for a comprehensive search of all available information and the identification of research gaps. The scoping review followed the guidelines provided in the Joanna Briggs Institute (JBI) Reviewer’s Manual and complied with the PRISMA extension for scoping reviews, PRISMA-ScR Guidelines [25].

To be included in this review papers needed to (a) report one or multiple phases of the cancer continuum, such as prevention, detection, diagnosis, treatment, survivorship or the additional phase proposed by the lead researcher; palliative care, or (b) discuss a common phenomenon present in multiple phases, or (c) discuss the perspectives of CALD migrants, clinicians, or migrants’ families and carers; (d) publish between 2000 and 2020 for currency; and (e) be written in English. The term ‘CALD migrants’ was used to define people who were born in countries where English is not a main language and who are also considered an ethnic or cultural minority in Australia. Whilst acknowledging the problematic nature of this terminology, it is currently in line with the Australian research landscape [26]. Qualitative, quantitative and mixed-method studies were included for comprehensiveness. No studies were excluded based upon quality. Studies were excluded if they did not report on CALD migrant populations, or their families, or the healthcare professionals interacting directing with them. Studies were excluded if they included non-malignant diseases or conditions. Studies were excluded if they grouped CALD populations with Australian-born participants, such as Aboriginal and Torres Strait Islander peoples. Studies published earlier than the year 2000 were excluded.

A comprehensive search strategy was developed in accordance with the JBI reviewer’s manual and the PRISMA ScR guidelines [25]. The search was designed to capture all relevant studies that combined the concepts of ‘CALD migrants’, ‘cancer care’, ‘access’ and ‘equity’ in the Australian healthcare system. The following databases were searched between 2000–2020:

- PubMed Central.
- CINAHL EBSCO.
- PsycInfo Ovid.
- Cochrane Library.
- Joanna Briggs Institute EDP Database.
- ProQuest Dissertation and Theses (Grey literature).

**Fig. 1:**
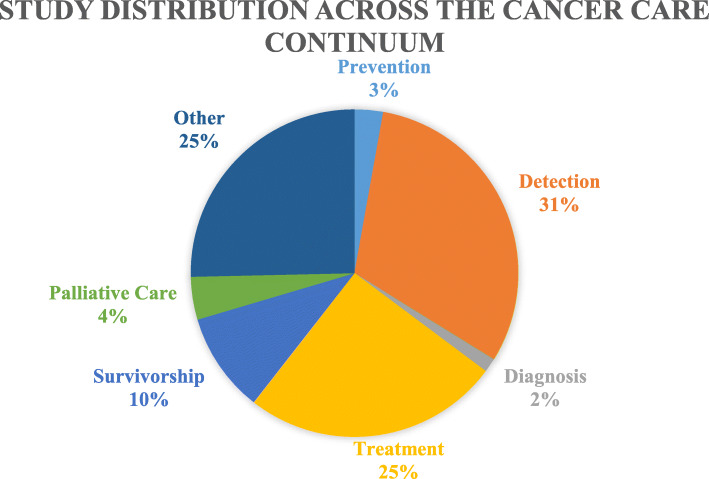
Study distribution across the cancer care continuum

After implementing the search protocol, the final search results were exported to Endnote and duplicates removed. The abstracts and full text articles identified through the search strategy were screened for relevance by the lead researcher and then by a second researcher, based on the inclusion and exclusion criteria. The search strategy is displayed in Fig. 1. Citations were managed using Endnote X9 and a Microsoft document. Discrepancies between authors were resolved through discussion. Included studies were charted using a data extraction form based on *JBI Reviewer’s Manual*, see in Table 1 [25].

**Table. 1:** Data extracted from studies

Data extracted	
Author	Location
Year	Sample Size
Cancer Care Continuum Stage Assessed	Population Group
Study Design	Methodology
Aims/Purpose	Key Findings

Data were extracted into an Excel spreadsheet and descriptively mapped to demonstrate the current landscape of the literature, rather than assessed analytically [25]. Frequency counts of key concepts, populations and characteristics of the current studies were completed. Data were disaggregated into the stages of the cancer care continuum to show the distribution of the studies across the continuum. The extracted data, shown in Table 2, were used to formulate tables and charts mapping the studies’ distribution based on geographic location, CALD group studied, sample size, year published, methodologies and main findings and to identify research gaps.

**Table. 2:** Sample of data extraction and charting process

Author	Year		CCC Stage	Study Design	Aims/Purpose	Location	Sample Size	Population Group	Methodology	Key Findings
Robotin, M C. et al.	2010		**Prevention**	Cohort study	Aiming to optimise the management of chronic Hepatitis B in at-risk populations via screening, surveillance and treatment. To prevent development of liver disease and liver cancer.	South-West Sydney, New South Wales, Australia	5, 800 local residents (hypothetical cohort)	Migrants from Hepatitis B endemic countries	Estimated numbers of CHB infections were derived from Australian Bureau of Statistics data. These figures were entered into a Markov model of disease progression, constructing a hypothetical cohort. The stages of CHB were calculated, as well as the primary and specialist healthcare resources needed annually by the cohort	1. There is a limited increase in GP consultations, a moderate increase in specialist consultations and a large increase in liver ultrasounds for this cohort annually2. New models of care are required in order to scale up the existing services available for CHB population
Schulz, T R. et al.	2014		Prevention	Cost-Benefit Analysis	To compare if screening for and eradication of Helicobacter pylori in immigrants reduces burden of gastric cancer	Melbourne, Victoria, Australia	N/A	Asymptomatic immigrants from high H. pylori prevalence areas	Nine different screening and follow-up strategies were compared with the current process of no screening	1. H. pylori screening and eradication can be an effective strategy for reducing rates of gastric cancer2. Data suggest that use of stool antigen testing is the most cost-effective approach
Aminisani, N. et al.	-2012		**Detection** (Screening)	Cohort Study	To assess the Cervical Cancer Screening behaviours of migrants, compared to Australian-born women	New South Wales, Australia	12, 541 migrants linked to 12, 143 Australian-born women	Middle Eastern or Asian-born women aged between 20–54 years	Year 2000 records of NSW Midwives Data Collection (country of birth) linked with Cervical Screening Register	1. Migrant women are less likely to participate in Cervical Screening than Australian-born women at the recommended interval2. Migrant women’s screening is less related to socio-economic status, smoking and parity as Australian-born women
Aminisani, N. et al.	2012		Detection (Screening)	Cohort Study	To assess the Cervical Screening behaviours of older migrant women, compared to Australian-born women	New South Wales, Australia	14, 228 migrants linked to 13, 939 Australian-born women	Middle Eastern or Asian-born women aged between 40–64 years	Year 2000–2001 records were compared to an age and area matched random sample of Australian-born women through the NSW Admitted Patients Data Collection and Cervical Screening Registers	1. Older women from the Middle East, North East and South East Asia appeared to have similar overall screening participation to that of Australian-born women2. Women from South Central Asia appeared less likely than Australian-born women to participate in cervical screening at the recommended interval
**Author**	**Year**		**CCC Stage**	**Study Design**	**Aims/Purpose**	**Location**	**Sample Size**	**Population Group**	**Methodology**	**Key Findings**
Aminisani, N. et al.	2012		Detection (Screening)	Cohort Study	To compare the rates of cervical cancer in migrant and Australian-born women after the introduction of Organised Cervical Screening	New South Wales, Australia	11,485 women	Women aged 15 + diagnosed with invasive cervical cancer between 1973–2008	Joinpoint regression was used to assess the annual percentage changes in incidence and mortality before and after the introduction of Organised Cervical Screening in 1991	1. Incidence and mortality rates fell post the introduction of Organised Cervical Screening for Australian-born, UK-born, Ireland-born women, and to a lesser extent woman from the Middle East, New Zealand, North Africa and Asian-born women.2. There was a rise in mortality found in women from a “rest of world” category, that may be explained by recent migration
Anaman, J A.et al.	2017		Detection (Screening)	Cross-sectional Survey	To compare the level of cervical screening uptake between refugee and non-refugee African immigrant women	Brisbane, Queensland, Australia	144 African Refugees, 110 African non-refugees	254 African women aged between 21–62, from 22 African countries	Chi-Square tests were used to compare demographic and health-related characteristics between refugee and non-refugee women. Multiple logistic-regression analyses were performed to assess the relationship between Pap-Smear testing and independent variables	1. Non-refugee women were significantly more likely to utilise pap-screening services than refugee women2. Significant predictors of screening uptake were work arrangement, parity, healthcare visit, knowledge and perceived susceptibility of cervical cancer
Anaman-Torgbor, J A. et al.	2017	Detection (Screening)		Qualitative Semi-Structured Interviews	To describe barriers and facilitators of cervical screening practices among African immigrant women living in Brisbane, Australia	Brisbane, Queensland, Australia	19 African Immigrant women;10 Refugee and 9 Non-Refugee	19 African immigrant women, aged between 21–65 years	Interviews were semi-structured and transcribed verbatim. They were analysed using interpretive thematic analysis.	1. Lack of knowledge about cervical cancer and Pap smear, the absence of warning signs, embarrassment, fear, concern about the gender of the service provider, lack of privacy, cultural and religious beliefs, and healthcare system factors were identified as barriers to screening
**Author**	**Year**		**CCC Stage**	**Study Design**	**Aims/Purpose**	**Location**	**Sample Size**	**Population Group**	**Methodology**	**Key Findings**
Cullerton, K. et al.	2016		Detection (Screening)	Cohort study	To understand the impact of education sessions on the knowledge and attitudes towards cancer screening.	Brisbane, Queensland, Australia	159 participants in 3 education sessions	7 CALD groups; Arabic-speaking, Bosnian, South Asian, Samoan and Pacific Island, Spanish-speaking, Sudanese and Vietnamese.	All individuals participated in culturally tailored cancer screening education program and a pre- and post-education evaluation measured changes in knowledge, attitudes and intention related to breast, bowel and cervical cancer and screening	1. Overall participants’ knowledge increased, some attitudes toward participation in cancer screening became more positive and intent to participate in future screening increased2. Culturally tailored education programs are effective in improving knowledge, attitudes about and intentions to participate in cancer screening
Kwok, C.et al.	2011	Detection (Screening)		Qualitative Interviews	To understand the barriers and facilitators to cervical cancer screening for Chinese-Australian women	New South Wales, Australia	18 Chinese-Australian women	Chinese-Australian women with no history of Cervical Cancer	18 women participated in qualitative interviews in their first language (Mandarin or Cantonese) and were analysed using content analysis	1. Knowledge of Cervical Cancer was low and few participants understood the purpose of screening2. Having a doctor’s recommendation was a strong motivator, as was having a female Chinese doctor and reminder letter

### Findings

**Fig. 2:**
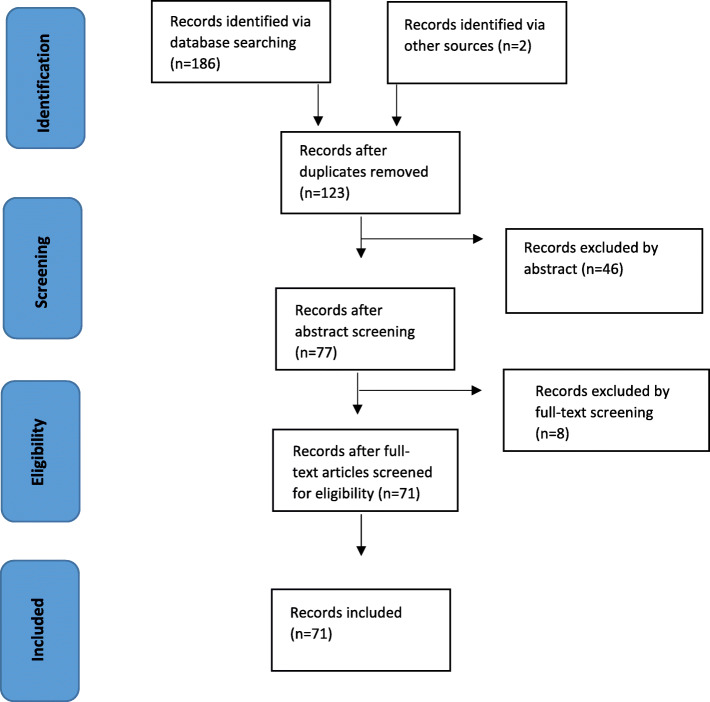
Search process illustrated in a PRISMA flowchart for scoping review

The search strategy identified 188 published studies. After removing duplicates there were 123 studies. After screening results by title and abstract there were 77 studies. After reading full-text articles, 71 studies met the inclusion criteria and were included in the final analysis. Details of the data extraction process are shown in Table 1. Figure 2. displays the distribution of the studies based on the stages of the cancer care continuum.

### Disparities in screening for prevention and detection

Two studies recommended targeted screening for migrants from hepatitis B and Helicobacter pylori endemic countries as a mode of cancer prevention [27, 28]. Those with chronic hepatitis B (CHB) are 6–12 times more likely to develop liver cancer and those from Helicobacter pylori (*H. pylori.*) endemic countries are at significantly higher risk of developing gastric cancers [27, 28]. Thus, through addressing disparities in CHB and *H. pylori*, cancer prevention can also be achieved for some migrant groups.

The detection phase of the cancer care continuum was measured through screening services and reported in twenty-two studies [29–50]. Six studies reported on the barriers and/or facilitators to screening services for cervical, breast and colorectal cancer [30–32, 39–41]. Common barriers identified were lack of screening knowledge, feelings of embarrassment, fear over the gender of health provider, privacy concerns, religious and cultural beliefs, language barriers, fatalistic views of cancer, screening not being promoted in community languages and location of services [30–32, 39, 40]. These findings largely perpetuated the individualistic, deficit perspective of barriers to care, with institutional barriers less often reported. Strong facilitators for screening were a doctor’s recommendation, having a female doctor of the same ethnic background, feeling understood by health providers and having strong social support [32, 39].

Ten studies focused on screening participation rates for breast, bowel and cervical cancers [33, 34, 36–38, 42, 44, 47–49]. A study of more than 24,000 women found that migrant women born in Middle Eastern and Asian regions were less likely to participate in cervical cancer screening than Australian-born women [37]. Similarly, women from African refugee backgrounds were significantly less likely to utilise cervical cancer screening services [49]. Factors associated with higher screening participation were length of residency in Australia, marital and employment status, flexibility of clinic hours, gender of the health practitioner, having a reminder system and accessible health information [42, 48].

### Diagnosis and treatment participation

Only one study examined the diagnostic pathway for CALD populations [51]. The LEAD protocol details a prospective observational cohort study to compare the diagnostic pathways for lung cancer between CALD and ‘Anglo-Australian’ populations, with no results available to date [51]. Eighteen studies analysed the treatment stage of the cancer care continuum [5, 12, 52–67]. Of these, four examined treatment coordination [52, 60, 63, 67]. A randomised control trial found calling women for appointment reminders in their preferred language significantly increased appointment and attendance rates and was more successful than translated reminder letters [52]. Shaw et al. (2016) described the experiences of cancer care coordination for Chinese, Arabic and Macedonian speaking patients, finding these migrants require additional assistance navigating the health system and information appropriate to their linguistic background [63].

Only three studies reported on equity in treatment outcomes [5, 12, 62]. Two of these compared the outcomes of Arabic, Chinese and Greek populations to that of Anglo-Australians and found the former had significantly worse health-related quality of life and higher incidence of clinical anxiety and depression [12, 62]. The third study examined Cantonese, Arabic and Mandarin speaking patients and found they experienced inequitable treatment quality due to the health services’ expectation of English proficiency and familiarity with the health system. This resulted in diminished understandings and explanations about cancer, treatment and the roles of specialists [5]. Two additional studies found CALD populations were significantly underrepresented, or not clearly represented in clinical trial research and participation [61, 64].

### Experiences of survivorship

Seven studies discussed the stage of survivorship for CALD populations [68–74]. Six were qualitative interviews or focus groups [68, 69, 71–74] and one cohort study [70]. Four of the qualitative studies retrospectively described experiences of inequity during cancer treatment. This displayed issues with incorrect interpreter usage, a greater need for information to manage illness and further explanations about tests and side effects prior to undergoing treatment [68, 69]. Similarly, Butow et al. (2013) found ‘immigrant cancer survivors’ were more likely to report unmet physical care or informational needs [70]. Ongoing cancer-related stressors in the survivorship stage, such as lack of culturally or linguistically specific survivorship information and resources, difficulty navigating health system and community entitlements and lack of appropriate caregiver information, were also reported [70, 72]. Studies further reported a reliance on family members and bilingual general practitioners for effective survivorship care coordination and a desire for acknowledgement of the diversity of survivorship experiences between CALD groups [72, 73].

### Palliative care

There were limited studies related to equity during palliative care for CALD populations and their families, with only three studies reporting on this [75–77]. In qualitative interviews with CALD patients and their families during the palliative care phase, Kirby et al. (2018) found issues during the transition to palliative care, such as poor communication about patient management and individuals not wishing to discuss death and dying directly, and highlighted the importance of cultural and spiritual needs [77]. A retrospective study examined a cohort of deceased CALD patients to examine the physical and psychological journey at end-of-life [76]. This found non-English speaking patients did not receive equitable assessment of physical symptoms at end-of-life [76]. Additionally, those who identified the need for an interpreter on admission, only 9 % accessed professional interpreters throughout their admission [76]. Additionally, poor documentation of cultural considerations was common, with post-death care of the patient’s body documented in only 20 % of cases [76].

### Communication

Poor communication across the cancer care continuum was a consistent theme, with eight studies discussing it directly [13, 78–84]. Communication was described as a significant barrier to equitable care, with migrants expressing feeling alone and misunderstood by health services [13, 80]. A lack of consistency with interpreters led to many feeling unable to understand medical instructions or communicate issues and concerns with health providers [13, 80]. A cohort study compared the oncology consultations of ‘immigrant’ patients, with and without interpreters, to that of Australian-born patients [78]. Findings showed doctors spoke less to immigrant patients with interpreters than to ‘Anglo-Australians’, spent proportionally less time discussing, summarising and informing on cancer-related issues, and tended to delay or omit more responses to immigrant patients [78]. This was thought to be due to the time constraints of repeating questions and responses through interpreters, as well as the incorrect assumption by some clinicians that ethnically diverse individuals prefer a paternalistic approach to communication. This was shown to be incorrect during post-consultation interviews [78].

### Patient and clinician perspectives

Three studies investigated the perspectives of clinicians directly [85–87] and four examined the perspectives of patients [88–91]. Clinicians reported limited culturally-appropriate translated resources, difficulty engaging appropriate interpreters, lack of funding, a culture of “learning on the job” and time constraints as significant structural barriers to providing equitable care for CALD populations [86, 87]. The importance of prioritising and developing quality relationships with CALD patients was highlighted in clinician focus groups as a facilitator of effective intercultural care [85]. Medical clinicians reported the tendency to refer complex culturally diverse patients to allied health or multicultural health workers, thus limiting their access to specialist oncology clinicians [85]. Patients reported wanting more information about cancer, as well as diagnostic and treatment options [89]. Lacking information was a persistent theme, with two further studies highlighting migrant women are significantly less likely to undergo breast reconstruction post-mastectomy, often due to a lack of information and counselling [88, 91]. Patients reported challenges communicating with health professionals and that many resources did not cater to those with limited English skills [89, 90].

## Discussion

This study presents a comprehensive review of the published literature regarding equity across the cancer care continuum for CALD migrant populations living in Australia. A key finding is the persistent focus within the literature on patient-sided barriers to care. This places a disproportionate level of burden on those who experience health inequities and obscures the structural, social and political processes that produce health inequities [92]. The lack of critique evident in the included studies shows researchers are also contributing to an individualistic, deficit perspective of health equity.

A key finding of this review is the role of health systems in creating and reinforcing cancer inequities. This is exemplified through pervasive communication problems across the cancer care continuum, a lack of culturally and linguistically appropriate cancer and treatment related information and a health system that is difficult for patients to navigate. In addition, clinicians report a context of inadequate support, resources and significant time constraints that restrict their ability to provide equitable care. These factors create a situation where CALD migrant populations are less informed about their health and treatment options, have difficulty communicating their concerns, find health services challenging to navigate and receive poorer quality care across many areas of the cancer care continuum [86, 87, 89, 93]. This demonstrates a culturally dominant model of care is not adequate in promoting equitable care for all populations and that targeted, culturally and linguistically responsive services, which support both patients and clinicians, are critical to equity [4].

Despite being a multilingual and multi-ethnic society, Australian health institutions have yet to take the necessary steps to move beyond the culturally dominant model of care. This model causes marginalisation of ethnic and cultural minority populations and reinforces assumptions and practices that lead to health inequities. This review also highlighted the need to provide further resources and training for clinicians, particularly in the development of the meaningful relationships needed to provide effective intercultural care [85]. It has been reported through focus-groups with clinicians that a lack of high-level interpreter services impedes their ability to assess patient symptoms and to develop intimate and trusting relationships with CALD patients [94]. This review also highlighted the need to expand health equity research into the field of palliative care. The limited Australian literature indicates significant challenges to achieving equitable and culturally appropriate palliative care [95]. Communication problems and a lack of attention to diverse values and practices at end-of-life have been highlighted as significant concerns [95]. Therefore, there is a need to investigate and establish new modes of caring that engage patients, families and communities [77].

This study revealed an uneven distribution of research spanning the cancer care continuum and an over-reliance on screening attendance rates as a measure of equity. This is significant because there are many cancers for which screening is not recommended or considered beneficial to patient outcomes [96]. Therefore, it can be argued that focus on other phases of the cancer care continuum, such as diagnostic pathways and treatment outcomes may have a greater influence on health equity for CALD migrant populations.

Few studies directly compared equity in access or outcomes, with most qualitative studies focusing on healthcare experiences or preferences of individuals. This is significant as there is growing acknowledgement that behavioral explanations of health inequities are inadequate and there is a need to examine the structural and institutional drivers of inequity [92]. For example, studies that describe patient-sided barriers to care, such as lack of English proficiency, are often describing institutional inaccessibility [5]. The expectation that patients speak English and can navigate the health system directly reflects the privileged positioning of majority groups and the marginalised positioning of others [97].

This review focused on equity within the Australian context, but highlights issues of global concern. With aging populations and an increased cancer burden in both OECD and emerging economies [7–9], the need for standardised equity indictors across the cancer care continuum is clear. This would allow for an expansion of research across the cancer care continuum and allow international health services to respond appropriately to the demographic and epidemiological changes associated with current and future migration flows [3, 7].

Due to the relatively small number of studies in each stage of the cancer care continuum and the heterogeneity of those studies, no conclusive findings can be taken from this review.

An important limitation to consider is that only studies published in English were included and as such many relevant studies in languages other than English may have been excluded. Additionally, most studies restricted populations to a small number of CALD groups, such as Chinese, Arabic and Greek speaking populations and therefore findings may not be generalisable across migrant groups.

## Conclusions

This study demonstrates that few studies have comprehensively assessed equity across the cancer continuum for CALD migrant populations in Australia. Within the current literature, there is a significant lack of critique examining the social, structural and institutional drivers of inequity. A further examination of power differentials, social positioning, marginalisation and the impact of majoritarian histories on health services will provide a deeper insight into the operationalisation of health equity. Research to date has placed a disproportionate burden on those who experience health inequities, rather than examining entrenched power differentials and institutional processes. It is necessary that health services shift their focus to the promotion of equity in order to become responsive to the diverse and changing needs of their populations.

## Data Availability

The datasets used and/or analysed during the current study are available from the corresponding author on reasonable request: b.scanlon@uq.net.au.

## Abbreviations

CALD: Culturally and linguistically diverse
CHB: Chronic Hepatitis B
H. pylori: Helicobacter pylori
